# Development of a validated UPLC-qTOF-MS Method for the determination of curcuminoids and their pharmacokinetic study in mice

**DOI:** 10.1186/2008-2231-21-11

**Published:** 2013-01-29

**Authors:** Mahendra K Verma, Ishtiyaq A Najar, Manoj K Tikoo, Gurdarshan Singh, Devinder K Gupta, Rajneesh Anand, Ravi K Khajuria, Subhash C Sharma, Rakesh K Johri

**Affiliations:** 1 PK/PD and Toxicology Division, CSIR-Indian Institute of Integrative Medicine, Canal Road, Jammu, India; 2 Analytical Chemistry Division (Instrumentation), CSIR-Indian Institute of Integrative Medicine, Canal Road, Jammu, India; 3 Biorganic Chemistry Division, CSIR-Indian Institute of Integrative Medicine, Canal Road, Jammu, India

## Abstract

**Background:**

A specific and sensitive UPLC-qTOF-MS/MS method has been developed for the simultaneous determination of curcuminoids. These Curcuminoids comprises of curcumin, a principal curcuminoid and other two namely, demethoxycurcumin, and bisdemethoxycurcumin obtained from rhizomes of *Curcuma longa* an ancient Indian curry spice turmeric, family (*Zingiberaceae*).

**Methods:**

These analytes were separated on a reverse phase C18 column by using a mobile phase of acetonitrile: 5% acetonitrile in water with 0.07% acetic acid (75:25 v/v), flow rate of 100 μL/min was maintained. The qTOF-MS was operated under multiple reaction monitoring (MRM) mode using electro-spray ionization (ESI) technique with positive ion polarity. The major product ions in the positive mode for curcuminoids were at m/z 369.1066, 339.1023 and 309.0214 respectively. The recovery of the analytes from mouse plasma was optimized using solid phase extraction technique.

**Results:**

The total run time was 5 min and the peaks of the compounds, bisdemethoxycurcumin, demethoxycurcumin and curcumin occurred at 2.06, 2.23 and 2.40 min respectively. The calibration curves of bisdemethoxycurcumin, demethoxycurcumin and curcumin were linear over the concentration range of 2–1000 ng/mL (r2, 0.9951), 2–1000 ng/mL (r2, 0.9970) and 2-1000 ng/mL (r2, 0.9906) respectively.

Intra-assay and inter-assay accuracy in terms of % bias for curcumin was in between −7.95to +6.21, and −7.03 to + 6.34; for demethoxycurcumin was −6.72 to +6.34, and −7.86 to +6.74 and for bisdesmetoxycurcumin was −8.23 to +6.37 and −8.47 to +7.81. The lower limit of quantitation for curcumin, demethoxycurcumin and bisdemethoxycurcumin was 2.0 ng/mL. Analytes were stable under various conditions (in autosampler, during freeze-thaw, at room temperature, and under deep-freeze conditions). This validated method was used during pharmacokinetic studies of curcumin in the mouse plasma.

**Conclusions:**

A specific, accurate and precise UPLC-qTOF-MS/MS method for the determination of curcumin, demethoxycurcumin and bisdemethoxycurcumin both individually and simultaneously was optimized.

## Background

*Curcuma longa L.* (*Zingiberaceae*) is a coloring agent, has been found to be a rich source of phenolic compounds, namely, curcuminoids (2-5%) [1]. *C. longa* consists of a mixture of three naturally occurring curcuminoids. Curcumin the principal curcuminoid (about 80%) and other two curcuminoids are demethoxycurcumin (about 12%) and bisdemethoxycurcumin (about 8%).

Curcuminoids are recognized for their broad spectrum biological activities and have been generally regarded as safe (GRAS) in foods or pharmaceuticals. Curcumin is widely used for coloring of foods like pickles and snacks.

Many pharmacological properties have been attributed to curcuminoids including anti-inflammatory and hepatoprotective activities [2], antioxidant and cholekinetic activities [3,4] and anti-protease activity [5,6]. In addition, apoptosis have been shown to induce in human cancer cells by the curcuminoids [7] and act as a chemopreventive agents for major types of cancer, including the stomach, lung, breast, prostate, colon and duodenal cancers, as well as leukemias [8-12] and display neuroprotective effects [13]. Curcumin has also been reported a more potent free radical scavenger than vitamin E [14].

It is also known for its potential use of curcumin in the treatment of infections such as human immunodeficiency virus (HIV) is also reported [15].

Quantification of the active metabolite, THC in plasma and urine by HPLC method has also been reported [16] and simultaneous quantification of diferuloylmethane and its metabolites in biological matrices has been reported by LC/MS/MS [17].

Hence, due to the immense biological importance [18] of curcumin and its analogues, there is a need for effective, rapid and more sensitive methods to monitor curcuminoids. Various HPLC methods are available in literature for determination of curcuminoids [19-29]. HPLC-MS methods also reported to provide quantitation of curcuminoids [30-32].

In recent times UPLC with qTof-MS is widely considered analytical technique for better quality data in terms of increased detection limits, and chromatographic resolution with greater sensitivity. This paper presents (i) a method for the simultaneous determination of curcuminoids by UPLC–qTOF-MS, and (ii) a pharmacokinetic study of curcumin in mice.

## Methods

### Material and methods

Curcumin, demethoxycurcumin and bis-demethoxycurcumin used as standards were isolated from the rhizomes of *C. longa* by the method already reported in literature. The isolated curcuminoids were identified on the basis of NMR and Mass spectral data. The purity of standards was >99%. All solvents/chemicals used were of HPLC grade and obtained from E-Merck, Mumbai, India. The HPLC grade water was obtained from a Water Purification System (Synergy UV, Millipore, USA).

### Instrumentation

A UPLC-qTOF-MS system (Synapt, Waters, USA, equipped with MassLynx acquisition software, version 4.1) was used. Experimental conditions were column, C-18 (50 × 2.1 mm); particle size, 1.7 μm; (Acquity, BEH); flow rate, 100 μL/min; mobile phase, acetonitrile: 5% acetonitrile in water with 0.07% acetic acid (75: 25 v/v), injection volume, 5 μL. The analyte infusion experiments were performed using an in-built syringe pump. A mass spectrometer with ESI interface was used for MS/MS analysis. ESI parameters were as follows: capillary voltage, 2.7 kV for positive mode; source temperature, 83°C; desolvation temperature, 200°C; cone gas flow, 50 L/h and desolvation gas flow, 550 L/h. The multiple reaction monitoring (MRM) mode was used to monitor the transition of curcumin m/z 391.0864 [M+Na], 369.1066 (M+H) to 285.0912, demethoxycurcumin at 339.1023 (M+H) to 255.0848 and of bisdemethoxycurcumin at m/z 309.0968 [M+H] to 225.0790.

### Preparation of reference, standard and quality control solutions

Reference solutions of curcumin (C) (stock I), demethoxycurcumin (DMC), (stock II) and bisdemethoxycurcumin (BDMC) (stock III) were prepared by weighing 5 mg of each compound. The quantities were transferred to 5 mL volumetric flasks, dissolved and diluted suitably with HPLC grade methanol. All the reference solutions (1 mg/mL) were covered with aluminium foil and sealed with paraffin film to avoid photodegradation and loss due to evaporation. Stock I, stock II and stoke III were mixed together, and diluted suitably with methanol. A 50-uL of this solution was used to spike blank mouse plasma samples (450 uL) to achieve 8 calibration standards (CAL STD) containing curcuminoids combination. CAL STD-1: curcuminoids, 2 ng/mL; CAL STD-2: 5 ng/mL each; CAL STD-3: 10 ng/mL each; CAL STD-4: 50 ng/mL each; CAL STD-5: 100 ng/mL each; CAL STD-6: 200 ng/mL each; CAL STD-7: 500 ng/mL each; and CAL STD-8: 1000 ng/mL each. Three quality control (QC) standards (LQC: 2 ng/mL; MQC: 450 ng/mL; HQC 900 ng/mL each of curcuminoids) were prepared and used to spike blank mouse plasma.

### Method validation procedures

The analytical method was validated to meet the acceptance criteria as per guidelines of the International Conference on Harmonization of Technical Requirements for Registration of Pharmaceuticals for Human Use (ICH). The specificity of the method was established by comparing blank plasma samples with those spiked with the analytes to find out interference from endogenous components. The CAL STD solutions were utilized for establishment of linearity and range (linear least-squares regression with a weighting index of 1/x). The precision and accuracy parameters were ascertained in LLOQ, LQC, MQC, and HQC samples (7 replicates each in 3 sets) on the same day and on 3 consecutive days. The intra-assay and inter-assay accuracy (% bias) of the method was determined from mean measured concentrations and nominal concentrations as follows: % bias = [(mean measured conc.−nominal conc.)/nominal conc.]×100. The intra-assay and inter-assay precision (% relative standard deviation or RSD) of the method was calculated from mean measured concentrations as follows: % RSD = (SD of mean measured conc./mean measured conc.)×100. The stability of analytes in plasma was investigated under following conditions: (a) 1 month storage at deep freeze (−80°C); (b) 3 consecutive freeze–thaw cycles from −20°C to room temperature; (c) 24 h storage at room temperature; and (d) short-term stability (of processed samples) at 10°C for 24 h in autosampler. After specified storage conditions, samples were processed and analyzed. The matrix effect was investigated by post extraction spike method. Peak area (A) of the analyte in spiked blank plasma with a known concentration (MQC) was compared with the corresponding peak area (B) obtained by direct injection of standard in the mobile phase. The ratio (A/B×100) is defined as the matrix effect.

### Sample preparation

The curcumin (C), demethoxycurcumin (DMC), and bisdemethoxycurcumin (BDMC*)* were recovered simultaneously from plasma using solid phase extraction (SPE) technique involving semi-automated vacuum chamber and vacuum pump (Supelco, USA). The various steps involved in the recovery procedure were: (a) conditioning of SPE cartridge (C18, 3 mL capacity, 100 mg bed, Samprep-Ranbaxy, Mumbai, India) with 1.0mLmethanol, followed by 1.0 mL water, (b) loading of diluted (1:4, v/v) plasma samples (1.0 mL) onto cartridge and drying under positive pressure, and (c) samples were washed with 2 mL of water followed by elution with 2 mL of methanol. The eluants were carefully collected in 2.0 mL capacity glass vials for direct analysis in UPLC–qTOF-MS system.

### Experimental animals

Swiss mice (22–30 g) were obtained from the Animal House of this Institute, and kept in regulated environmental conditions (temperature: 25 ± 2°C, humidity: 60 ± 5%, 12 h dark/light cycle). Animals were fed on standard pelleted diet (Ashirwad Industries, Chandigarh, India) and water was provided ad libitum. Animal experiments were approved by Institutional Ethics Committee. Animals were fasted overnight before the experiment and segregated into different groups for the sample collections at different time intervals. All these animals were administered with curcumin (100 mg/kg, p.o.). Blood samples were collected in pre-heparinized glass tubes at different time intervals post dosing (0–24 hr). Blood samples were centrifuged (5000 rpm; 10 min at 20°C) to separate the plasma.

### Pharmacokinetics

Concentration-time curves for Concentration–time curves were established for curcumin from the treated mice and used for the determination of pharmacokinetic parameters such as peak plasma concentration (Cmax), peak time (Tmax), extent of absorption (AUC), half-life (t1/2), clearance (Cl), and volume of distribution (Vd) by a non-compartmental analysis using PK Solutions Version 2.0; Summit Research Services, USA.

## Results

### UPLC–qTOF-MS/MS analysis

**Figure 1:**
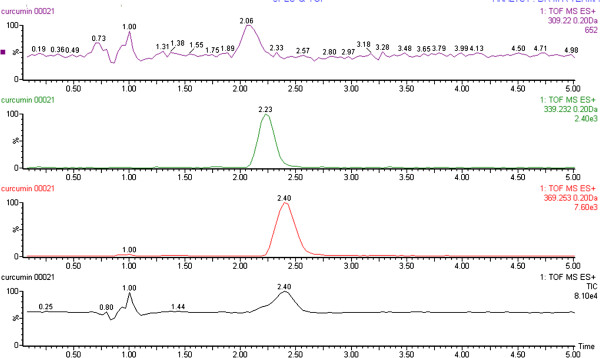
**Typical UPLC-qTOF-MS/MS chromatograms showing Curcuminoids.** 1 **A**. Extracted ion chromatogram (EIC) of bisdemethoxycurcumin. 1 **B**. Extracted ion chromatogram (EIC) of demethoxycurcumin. 1 **C**. Extracted ion chromatogram (EIC) of curcumin. 1 **D**. Total ion chromatogram (TIC) of Curcuminoids.

**Figure 2:**
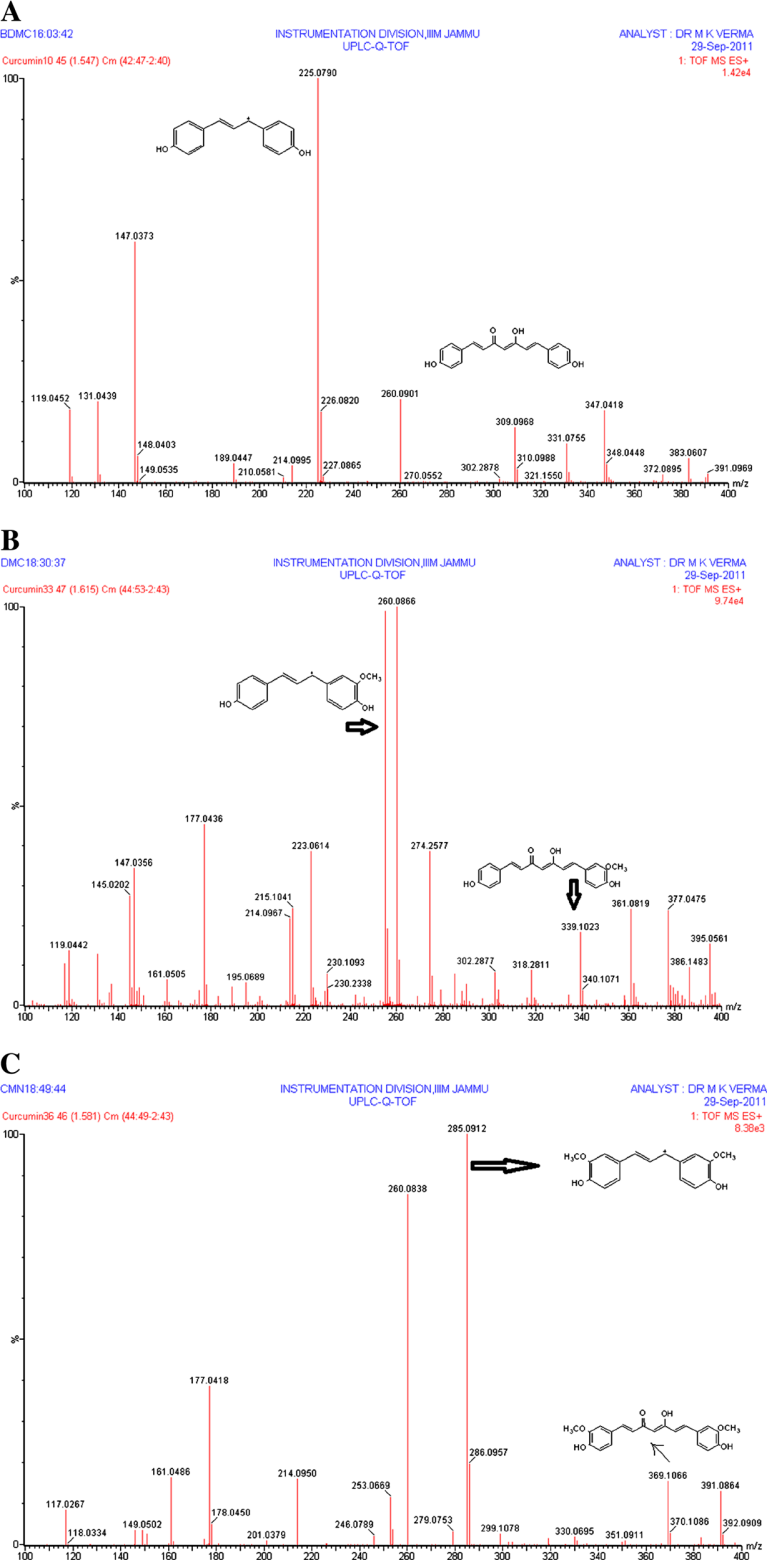
**2 A - Product ion spectra of bisdemethoxrcurcumin showing fragmentation transitions.** 2 **B** - Product ion spectra of demethoxrcurcumin showing fragmentation transitions. 2 **C** - Product ion spectra of curcumin showing fragmentation transitions.

Optimum chromatographic separation of curcuminoids was achieved by acetonitrile: 5% acetonitrile in water with 0.07% acetic acid (75:25 v/v). Flow rate of 100 μL/min was maintained. All the analytes were added simultaneously in the samples and the resulting chromatograms showed a retention time of 2.06, 2.23 and 2.40 min for bisdemethoxycurcumin, demethoxycurcumin and curcumin respectively (Figure 1A, B & C). A full scan in positive ion mode was used for all the analytes. During direct infusion, the mass spectra of the major product ions in the positive mode for bisdemethoxycurcumin m/z 309.0968 [M+H]^+^ to the product ion 225.0790 (Figure 2A) demethoxycurcumin m/z 339.1023 (M+H) to 255.0848 (Figure 2B) and of curcumin m/z 391.0864 [M+Na], 369.1066 (M+H) to 285.0912 (Figure 2C).

### Method validation

#### Specificity

The method was found to be specific: Extracted blank plasma when compared with plasma samples spiked with curcuminoids did not show any interference at the respective retention times of each analyte.

### Linearity and range

The calibration curves of bisdemethoxycurcumin, demethoxycurcumin and curcumin were linear over the concentration range of 2–1000 ng/mL (r2, 0.9951), 2–1000 ng/mL (r2, 0.9970) and 2-1000 ng/mL (r2, 0.9906) respectively.

### Accuracy and precision

The combined recovery of curcumin, demethoxycurcumin and bisdemethoxycurcumin was carried out in LLQC, LQC, MQC and HQC samples. The recovery (mean ± S.E.) of curcumin was 93.2% ± 4.1 (from LLQC), 95.6% ± 3.9 (from LQC), 96.2% ± 3.2 (from MQC), and 93.4% ± 2.9 (from HQC). The recovery (mean ± S.E.) of demethoxycurcumin was 92.8% ± 4.3 (from LLQC) 94.3% ± 3.8 (from LQC), 91.7% ± 3.3 (from MQC), and 91.5% ± 2.7 (from HQC). The recovery (mean ± S.E.) of bisdemethoxycurcumin was 89.9% ± 6.2 (from LLQC), 93.3% ± 5.2 (from LQC), 91.6% ± 3.2 (from MQC), and 90.5% ± 2.6 (from HQC). The intra-assay and inter-assay accuracy in terms of % bias were given in Table 1.

**Table 1:** Accuracy (% bias) data

**Compound**	**Nominal conc. (ng/mL)**	**Intra -assay**			**Inter-assay**		
		**Set 1**	**Set 2**	**Set 3**	**Set 1**	**Set 2**	**Set 3**
Curcumin	**2**	+5.81	−7.95	−7.02	+6.34	+5.38	−5.86
	**6**	+5.98	−6.29	+6.21	−7.03	+4.39	+5.39
	**450**	−4.23	+5.09	− 3.86	−5.61	+5.94	4.79
	**900**	+3.84	−4.72	−5.37	+4.67	−5.38	−5.01
DMC	**2**	+6.34	−5.81	−6.62	+6.74	+5.89	−5.06
	**6**	+5.68	−6.72	+6.04	−7.86	+4.95	+6.21
	**450**	−4.73	+5.89	− 5.31	−5.88	+5.04	−5.86
	**900**	+5.09	−4.38	−5.66	+5.17	−6.72	−4.71
BDMC	**2**	+6.37	−8.23	−7.91	+6.73	+7.81	−6.54
	**6**	+ 5.28	−6.98	+7.41	−8.47	+6.39	+5.86
	**450**	−6.29	+5.73	− 6.06	−6.80	+5.68	− 6.89
	**900**	+5.87	−6.32	−6.98	+5.69	−4.63	− 7.08

Intra- assay and inter-assay precision (% RSD) were presented in Table 2.

**Table 2:** Precision (% RSD) data

**Compound**	**Nominal conc. (ng/mL)**	**Intra -assay**			**Inter-assay**		
		**Set 1**	**Set 2**	**Set 3**	**Set 1**	**Set 2**	**Set 3**
Curcumin	**2**	8.31	7.34	7.93	8.59	6.57	7.81
	**6**	6.38	7.51	8.76	7.77	8.32	6.68
	**450**	4.68	6.39	5.51	5.31	6.07	5.85
	**900**	2.94	3.89	4.79	5.28	4.79	5.53
DMC	**2**	9.27	8.86	9.65	9.85	8.89	9.41
	**6**	7.41	6.09	7.38	7.05	6.59	9.77
	**450**	5.24	4.84	6.07	6.18	7.04	6.17
	**900**	4.41	4.79	6.06	4.69	5.31	4.99
BDMC	**2**	11.37	9.98	10.57	9.23	10.88	10.54
	**6**	8.68	7.98	9.07	9.35	8.47	9.86
	**450**	5.86	7.79	8.95	6.32	5.89	5.49
	**900**	4.46	5.32	5.48	3.69	4.79	6.24

The accuracy and precision of the method were within the acceptable limits of ±15%.

### Lower limit of quantitation (LLOQ)

The LLOQ for curcumin, demethoxycurcumin and bisdemethoxycurcumin were 2.0 ng/mL.

### Stability

The stability of the analytes in plasma was investigated in LQC and HQC samples. The recoveries of the analytes after one month (storage stability), after 1, 2 and 3 cycles of freeze–thaw and after 24 h (stability at room temp.) relative to that at time zero are summarized in Table 3.

**Table 3:** Stability data

**Condition**	**CMN**		**DMC**		**BDMC**	
	**LQC**	**HQC**	**LQC**	**HQC**	**LQC**	**HQC**
**Recovery (ng) after storage (−80°C)**	5.92 ±0.141	888.974 ± 1.876	5.90 ±0.161	885±1.948	5.71±0.209	874 ±2.375
** 0 month**						
** 1 month**	5.81±0.165 (98.14%)	853.41 ± 1.451 (95.99%)	5.78±0.178 (97.97%)	861±1.381 (97.29%)	5.57±0.393 (97.55%)	845 ±3.058 (96.68%)
**Recovery (ng) after freeze thaw cycles**	5.92 ± 0.141	888.974 ± 1.876	5.90± 0.161	885±1.948	5.71±0.209	874 ±2.375
** Cycle 0**	5.82±0.186	878.413±	5.84±0.181	869±1.768	5.65±0.219	870 ±2.435
** Cycle 1**	(98.31%)	1.381 (98.81%)	(98.98%)	(98.19%)	(98.94%)	(99.54%)
	**5.76**±0.314	871.274±	5.81± 0.173	865±1.549	5.63±0.179	864 ±2.021
** Cycle 2**	(97.29%)	1.576 (98.00%)	(98.47%)	(97.74%)	(98.59%)	(98.85%)
	**5.62** ±0.372	852.560± 1.732	5.78± 0.196	861±1.598	5.60±0.247	859 ±2.564
** Cycle 3**	(94.93%)	(95.90%)	(97.96%)	(97.28%)	(98.07%)	(98.28%)
**Recovery (ng) after storage at room temp.**	5.92 ± 0.141	888.974 ± 1.876	5.90± 0.161	885±1.948	5.71±0.209	874 ±2.375
0 h	5.62± 0.198	832.231 ±	5.77± 0.293	829±1.321	5.37±0.901	821 ±1.967
24 h	(94.93%)	1.451 (93.61%)	(97.79%)	(93.67%)	(94.04%)	(93.93%)
**Recovery (ng) after storage in auto sampler**	5.92 ± 0.141	888.974 ± 1.876	5.90± 0.161	885±1.948	5.71±0.209	874 ±2.375
** 0 h**	5.80± 0.219	872.136 ±	5.77± 0.293	858±1.973	5.56±0.214	854 ±3.057
** 24 h**	(97.97%)	2.151 (98.10%)	(97.79%)	(96.95%)	(97.37%)	(97.71%)

### Matrix effect

The matrix effect (A/B×100) for Curcumin was 96.78% (% RSD: 3.14; n = 5), and for DMC it was 97.31% (% RSD: 4.05; n = 5) and for BDMC it was 96.13% (% RSD: 3.89; n=5) Percent RSD < 5 suggested that the method was free from matrix effect.

### Pharmacokinetics

**Figure 3:**
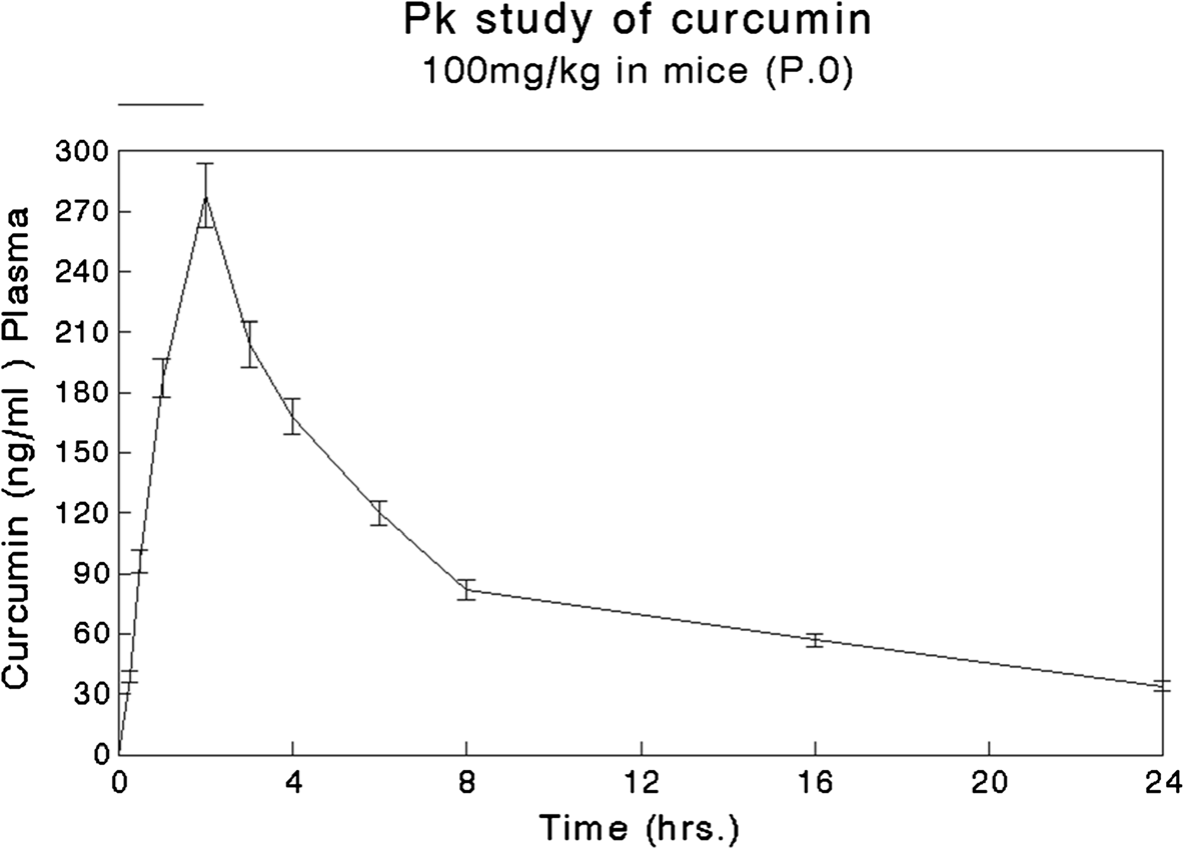
Plasma concn. vs. time curves of curcumin (100 mg/kg, p.o).

Concentration vs. time profile of Curcumin and pharmacokinetic parameters (Figure 3, Table 4). Each time point is mean±SE (n = 6). For details refer Section 1.6.

**Table 4:** Pharmacokinetic parameters (curcumin)

	
AUC _0-∞ (_ng*hr/ml)	2650.9± 13.2
C _max_ (ng/ml)	278.0± 18.6
T _max_ (hr)	2.0
Half Life (hr.)	10.1±0.96
Clearance (L/hr)	46.125±2.39
Vd (L/kg)	502.06±23.2
MRT (hr.)	18.3±1.21

## Discussion

**Figure 4:**
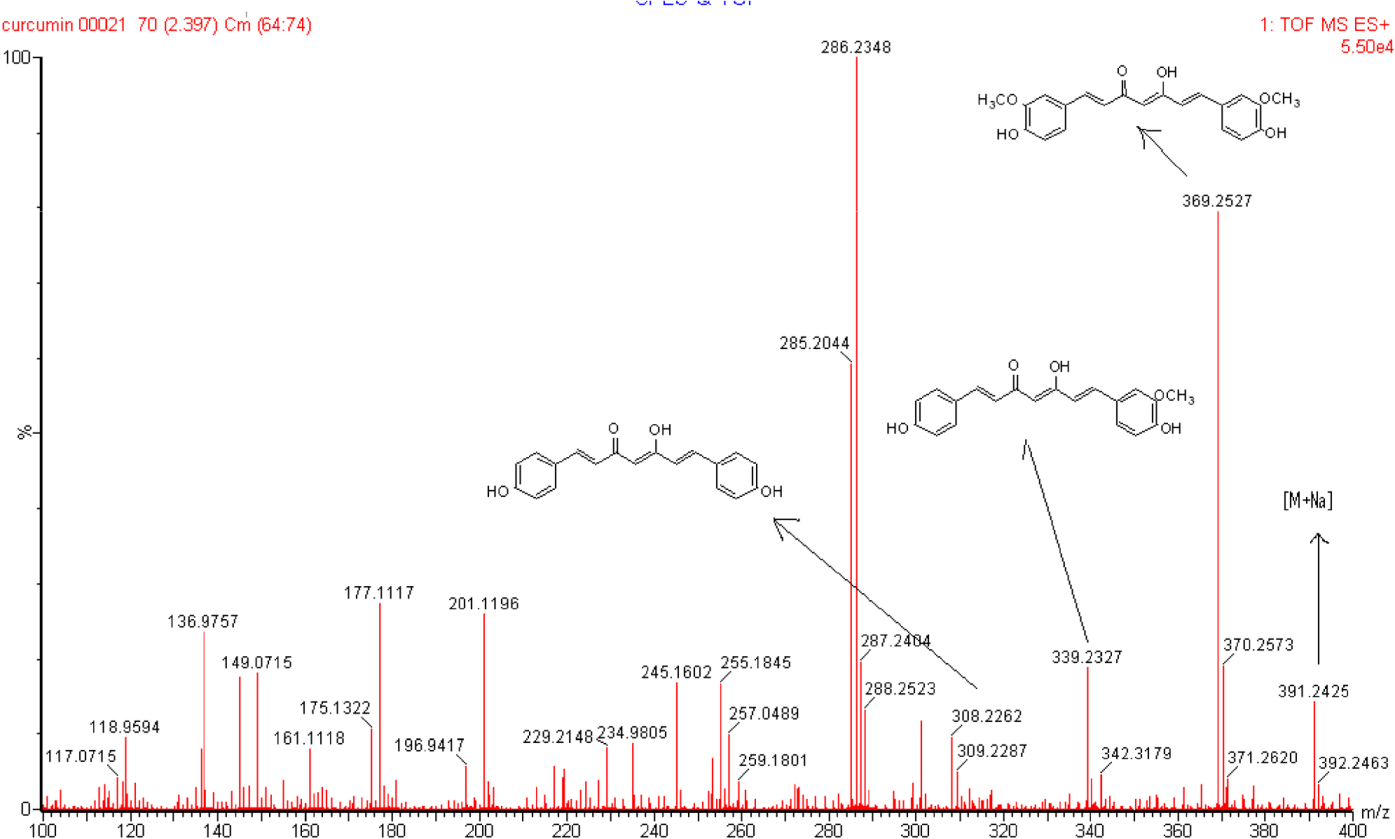
The mass spectrum of curcuminoids in combination showing the molecular ion peaks, chemical structures and molecular weights.

Three curcuminoids were used as a standard in the present study showed separate peaks in the extracted ion chromatogram (EIC) at 2.06, 2.23 and 2.40 min for bisdemethoxycurcumin demethoxycurcumin and Curcumin respectively. The bisdemethoxycurcumin demethoxycurcumin and Curcumin, were also appeared in the total ion chromatogram TIC. These three curcuminods were also shown together in the ESI spectra (Figure 4).

A method for the determination of curcumin, demethoxycurcumin and bisdemethoxycurcumin by UPLC-qTOF-MS/MS has not been reported, prior to this investigation, in which curcuminoids have been quantified on the basis of their major fragment. The major product ions observed in the positive ion ESI spectra curcumin m/z 391.0864 [M+Na], 369.1066 (M+H) to 285.0912, demethoxycurcumin at 339.1023 (M+H) to 255.0848 and of bisdemethoxycurcumin at m/z 309.0214 [M+H]^+^ to the production 225.0790. The quantification of the analytes was achieved by using MRM which makes the proposed method most acceptable.

Previously reported HPLC-UV methods for the quantification and determination of curcuminoids have several disadvantages, such as unsatisfactory separation times (needs more analysis time), poor resolution and complicated solvent mixtures with gradient elution. These methods are not selective, rapid, so a time-consuming pretreatment of a sample, or complicate gradient elution is required.

We have developed a simple, reliable and an isocratic UPLC-qTOF-MS/MS method which require only binary solvent system containing water and acetonitrile. This method has shown high degree of simplicity, accuracy, sensitivity, reproducibility and also provides short analysis time (5 min.). In the proposed method the linearity was in the range between 2 ng/mL to 1000 ng/mL which makes the method most suitable for the trace quantification of analytes. This method can also be used for the quantification of individual curcuminoids for routine analysis.

The method was validated in terms of specificity, accuracy, precision, sensitivity and stability of the analytes, and utilized for the determination of curcumin, demethoxycurcumin and bisdemethoxrcurcumin either individually or simultaneously in plasma (mice). After oral administration curcumin could be quantified only up to 24 h of sampling time. A pharmacokinetic parameters from plasma concentration-time data usually involves the maximum (peak) plasma drug concentrations (*C*_max_) and the area under the plasma concentration –time curve (AUC). The plasma drug concentration increases with the rate of absorption; therefore the most widely used general index of absorption is *C*_max_. AUC is another reliable measure for the extent of absorption. It is directly proportional to the total amount of unchanged drug that reaches systemic circulation.

## Conclusions

A specific, accurate and precise UPLC-qTOF-MS/MS method for the determination of curcumin, demethoxycurcumin and bisdemethoxycurcumin both individually and simultaneously was optimized. Pharmacokinetic study of curcumin was carried out by using this validated method.

## Competing interests

The authors declare that they have no competing interests.

## Authors’ contributions

MK, SC and RK conceived and designed the study and prepared the manuscript. MK, SC, IA, GD and MT carried out all the experimental work and statistical analysis and helped to draft the manuscript. MK carried out the UPLC –Q-TOF-MS studies, DK isolated the compounds by using column chromatography studies and compounds were characterized by MK, DK, RA and RKK. All authors read and approved the final manuscript.
